# Procedural and documentation variations in intravenous infusion administration: a mixed methods study of policy and practice across 16 hospital trusts in England

**DOI:** 10.1186/s12913-018-3025-x

**Published:** 2018-04-10

**Authors:** Dominic Furniss, Imogen Lyons, Bryony Dean Franklin, Astrid Mayer, Gillian Chumbley, Li Wei, Anna L. Cox, Jolien Vos, Galal Galal-Edeen, Ann Blandford

**Affiliations:** 1 UCL Interaction Centre, 66-72 Gower Street, London, WC1E 6BT UK; 2 Centre for Medication Safety and Service Quality, Imperial College Healthcare NHS Trust, Charing Cross Hospital, Fulham Palace Road, London, UK; 3 Research Department of Practice and Policy, UCL School of Pharmacy, Mezzanine Floor, BMA House, Tavistock Square, London, UK; 4 Royal Free London NHS Foundation Trust & UCL Medical School, Pond Street, London, NW3 2QG UK; 5 Pain Management Centre, Imperial College Healthcare NHS Trust, London, UK; 6 Information Systems Department, Faculty of Computers and Information, Cairo University, Cairo, Egypt

**Keywords:** Intravenous infusions, Medication errors, Mixed methods, Organizational standards, Observational study, Policy, Practice, Safety management

## Abstract

**Background:**

Procedural and documentation deviations relating to intravenous (IV) infusion administration can have important safety consequences. However, research on such deviations is limited. To address this we investigated the prevalence of procedural and documentation deviations in IV infusion administration and explored variability in policy and practice across different hospital trusts.

**Methods:**

We conducted a mixed methods study. This involved observations of deviations from local policy including quantitative and qualitative data, and focus groups with clinical staff to explore the causes and contexts of deviations. The observations were conducted across five clinical areas (general medicine, general surgery, critical care, paediatrics and oncology day care) in 16 National Health Service (NHS) trusts in England. All infusions being administered at the time of data collection were included. Deviation rates for procedural and documentation requirements were compared between trusts. Local data collectors and other relevant stakeholders attended focus groups at each trust. Policy details and reasons for deviations were discussed.

**Results:**

At least one procedural or documentation deviation was observed in 961 of 2008 IV infusions (deviation rate 47.9%; 95% confidence interval 45.5–49.8%). Deviation rates at individual trusts ranged from 9.9 to 100% of infusions, with considerable variation in the prevalence of different types of deviation. Focus groups revealed lack of policy awareness, ambiguous policies, safety and practicality concerns, different organisational priorities, and wide variation in policies and practice relating to prescribing and administration of IV flushes and double-checking.

**Conclusions:**

Deviation rates and procedural and documentation requirements varied considerably between hospital trusts. Our findings reveal areas where local policy and practice do not align. Some policies may be impractical and lack utility. We suggest clearer evidence-based standardisation and local procedures that are contextually practical to address these issues.

**Electronic supplementary material:**

The online version of this article (10.1186/s12913-018-3025-x) contains supplementary material, which is available to authorized users.

## Background

Intravenous (IV) medication, which includes both infusions and bolus doses, is associated with high rates of preparation and administration errors [1, 2]. A systematic review suggests IV doses are associated with five times more errors than non-IV doses [3]. IV infusion administration is thus a serious safety concern [4], with numerous policies and procedures in place at every hospital with the aim of reducing the risk of harm.

In 2007, recommendations were made in a Patient Safety Alert for England and Wales to reduce errors in injectable medicines, including risk-assessing procedures and products, reviewing protocols, providing technical information, competency-based training, and conducting an annual medicines management audit [5]. It highlighted how procedures should be “clearly documented, reflect local circumstances and describe safe practice that all practitioners can reasonably be expected to achieve. Patient safety incidents commonly result where procedures are absent, incomplete or where staff do not follow written procedures due to a lack of awareness, insufficient knowledge or because they do not agree with them and routinely violate them” ([5], page 3). Ten years on from this alert, it is not known whether and how health care organisations have adapted their procedures in light of this advice or how well these procedures are adhered to in practice.

Research into procedural and documentation deviations of IV infusion administration is also limited. Husch et al. [6] included procedural and documentation errors in their study of 426 IV infusions in a US hospital; this study found two of the most prevalent error types to be no rate on the additive label, affecting 46% of infusions, and patient identification (ID) issues, affecting 13% of infusions. Schnock et al. [7] used a similar method across ten US hospitals to examine 1164 IV infusions and reported 60% of infusions with an additive label that deviated from policy, 35% of infusions where giving sets were not labelled according to policy, and 0.2% of infusions where the patient had no ID wristband. No studies have investigated procedural and documentation deviations in the UK and none have explored the surrounding context or possible reasons for the discrepancies identified.

This study therefore aims to investigate the prevalence of procedural and documentation deviations related to IV infusion administration as part of a larger study of IV medication administration practices across 16 NHS trusts in England [4], and to explore variability in policy and practice across these trusts.

## Methods

### Study design

We used a mixed methods exploratory sequential design [8]. An adapted quantitative point-prevalence method, based on previous studies in this field, [6, 7] focused on ‘what’ and ‘how many’ deviations occurred in practice. This was followed by debriefs with observers and focus groups with key stakeholders to explore ‘why’ they occurred. The study protocol was published previously [4] and approved by an NHS Research Ethics Committee (14/SC/0290).

### Study setting and sample

We recruited 16 trusts (NHS organisations) in England. Using a purposive sampling strategy we selected hospitals that had a diverse range of IV infusion practices and differed in terms of type, size, geographic location, patient safety metrics, and use of infusion devices and smart pump technology [4]. We conducted observations in five clinical areas: general medicine, general surgery, critical care, paediatrics and oncology day care. Eight trusts conducted observations in all five areas; five conducted the study in the first three areas only; two specialist children’s trusts collected paediatric data only; and one trust collected oncology day care data only. Most trusts conducted the study at one hospital; the trust that collected only oncology day care data did so at specialist cancer units on three different hospital sites: 18 hospital sites were therefore included in total.

### Observational data collection

Each participating trust had a pair of observers (typically a nurse and a pharmacist) employed in that trust who collected data for about 1 day in each clinical area. Standardised training was provided to all observers. Observers compared each infusion that was running at the time of observation against the prescription, local policies and guidance [4]. Infusion preparation and set up were not observed. Observers also consulted clinical staff as needed to better understand the context of any deviations. Infusions included any medication, fluids, blood products and nutrition administered via an IV infusion, including patient-controlled analgesia. Bolus doses were excluded, except where a prescribed bolus was given as an infusion, or vice versa. Infusions that were completed were excluded even if connected to the patient. Patients were not included in the study if, for example, they were in isolation due to infection risks, were receiving care that would have required interruption at the time of observation, or were away from the clinical area (such as to have an X-ray).

Observers recorded data using a standardised paper form, which was then entered into a secure web-based data collection tool [9] and checked for consistency by the research team. No patient identifiable data were recorded. Suspected medication and documentation errors were raised with clinical staff so they could be corrected if needed; usual hospital practices for reporting errors were then followed.

### Identifying procedural or documentation deviations

We specified four types of procedural and documentation deviations a priori [4]: 1) giving sets not labelled appropriately; 2) documentation of administration inaccurate or incomplete; 3) infusion additive labels missing, incomplete or incorrect; and 4) patient ID wristbands missing or with incorrect, illegible or missing information. For each of these deviations, we collected both quantitative and qualitative data. Observers were encouraged to record any other deviations that did not fit within these types. We identified two further types of deviation where policies varied among trusts: 5) prescription and administration of IV flushes, and 6) procedures for the double checking of medication. For these latter categories, we present qualitative data only because they were not observed systematically but emerged in the debriefs and focus groups.

Multiple deviations could occur in each infusion, e.g. a missing giving set label and an incomplete additive label could affect the same infusion. We here report data on procedural and documentation deviations. Data on deviations relating to medication administration will be reported in a separate paper.

### Debrief and focus group data collection

Following observational data collection at each trust, a report was drafted summarising that trust’s data. This was presented at a debrief meeting with the observers, providing an opportunity to clarify aspects of the policies, practices and deviations observed. These clarifications sometimes led to updates to the data; e.g., one site realised that giving set labels in their critical care unit were not compliant with their policy because they did not include the date the infusion was set up; another site initially included some infusions that were completed but were still connected to the patient so these were subsequently excluded. Focus groups were then conducted with other local stakeholders to contextualise the findings, explore details of policies and practices and reasons for deviations, and discuss implications of the findings. Debriefs and focus groups were audio recorded and transcribed verbatim. Guides for debrief and focus group sessions can be found as a supplementary file to this paper (i.e., Additional file 1).

### Data management and analysis

**Fig. 1:**
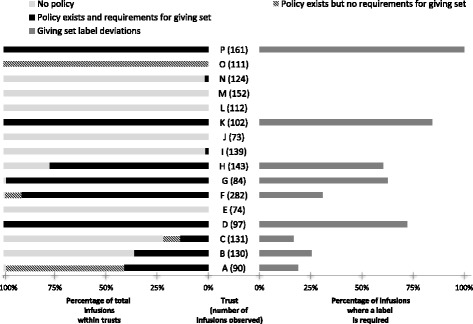
Variation in giving set label policy and deviations where a label is required among trusts

Procedural and documentation deviation rates were calculated as the proportion of infusions with at least one deviation, using total opportunities for error (TOE: total number of doses administered, plus any omitted doses) as the denominator (see Tables 1, 2, 3). However, policy requirements were particularly unevenly distributed in some areas. Here split charts were used to present the variability of local policy requirements (left hand side), and deviation rates calculated as the proportion of infusions with at least one deviation using TOE as the total number of doses administered where local requirements applied as the denominator (right hand side) (see Figs. 1 and 3). Debrief and focus group data were analysed inductively, and used to contextualise the quantitative data and provide explanatory detail about issues within and across trusts.

**Table 1:** Overall procedural and documentation deviations by trust

Trust	All trusts	Trust A	Trust B	Trust C	Trust D	Trust E	Trust F	Trust G	Trust H	Trust I	Trust J	Trust K	Trust L	Trust M	Trust N	Trust O	Trust P
Number of infusions	2008	90	130	131	97	74	282	84	143	139	73	103	114	152	124	111	161
Overall number of infusions with one or more deviation	961 (47.9%)	33 (36.7)	69 (53.1)	55 (42)	76 (78.4)	34 (45.9)	84 (29.8)	53 (63.1)	90 (62.9)	68 (48.9)	13 (17.8)	96 (93.2)	22 (19.3)	15 (9.9)	77 (62.1)	15 (13.5)	161 (100)

Numbers in brackets refer to percentage of infusions with at least one deviation

**Table 2:** Documentation deviations by trust

Trust	All trusts	Trust A	Trust B	Trust C	Trust D	Trust E	Trust F	Trust G	Trust H	Trust I	Trust J	Trust K	Trust L	Trust M	Trust N	Trust O	Trust P
Number of infusions	2008	90	130	131	97	74	282	84	143	139	73	103	114	152	124	111	161
Documentation of the administration	335 (16.7%)	10 (11.1)	24 (18.5)	32 (24.4)	7 (7.2)	27 (36.5)	19 (6.7)	4 (4.8)	18 (12.6)	38 (27.3)	7 (9.6)	23 (22.3)	13 (11.4)	7 (4.6)	41 (33.1)	10 (9.0)	55 (34.2)
Start time incorrectly or not documented	269	8 (8.9)	24 (18.5)	26 (19.8)	1 (1.0)	22 (29.7)	13 (4.6)	3 (3.6)	12 (8.4)	36 (25.9)	2 (2.7)	22 (21.4)	10 (8.8)	4 (2.6)	33 (26.6)	3 (2.7)	50 (31.1)
Nurse’s signature missing	103	8 (8.9)	1 (0.8)	23 (17.6)	1 (1.0)		2 (0.7)	3 (3.6)	5 (3.5)	8 (5.8)	1 (1.4)	10 (9.7)	8 (7.0)		25 (20.2)		8 (5.0)
Other	75	1 (1.1)	1 (0.8)	4 (3.1)	5 (5.2)	6 (8.1)	9 (3.2)	2 (2.4)	3 (2.1)	2 (1.4)	4 (5.5)	2 (1.9)	6 (5.3)	3 (2.0)	15 (12.1)	7 (6.3)	5 (3.1)
Documentation of the medication order^a^	36 (1.8%)	2 (2.2)	5 (3.8)	4 (3.1)		3 (4.1)	5 (1.8)		1 (0.7)	9 (6.5)	1 (1.4)		1 (0.9)		5 (4.0)		

Numbers in brackets refer to percentage of infusions with at least one deviation

^a^Category added during analysis phase based on wider reported deviations to do with an incorrect, incomplete, poorly documented or ambiguous medication order

**Table 3:** Procedural deviations by trust

Trust	All trusts	Trust A	Trust B	Trust C	Trust D	Trust E	Trust F	Trust G	Trust H	Trust I	Trust J	Trust K	Trust L	Trust M	Trust N	Trust O	Trust P
Number of infusions	2008	90	130	131	97	74	282	84	143	139	73	103	114	152	124	111	161
Giving set not labelled correctly	537 (26.8%)	7 (7.8)	12 (9.2)	3 (2.3)	70 (72.2)		79 (28.0)	52 (61.9)	67 (46.9)			86 (83.5)					161 (100)
No label (where required)	496	7 (7.8)	12 (9.2)	3 (2.3)	70 (72.2)		74 (26.2)	52 (61.9)	64 (44.8)			86 (83.5)					128 (79.5)
Incomplete or incorrect label	41						5 (1.8)		3 (2.1)								33 (20.5)
Start date	37						3		1								33
Discard date	2								2								
Name of drug/fluid	2						2										
Other	5						2		2								1
Additive label missing or incorrect	219 (10.9%)	18 (20.0)	24 (18.5)	22 (16.8)	9 (9.3)	6 (8.1)	12 (4.3)	1 (1.2)	8 (5.6)	23 (16.5)	4 (5.5)	27 (26.2)	3 (2.6)	8 (5.3)	37 (29.8)	2 (1.8)	15 (9.3)
No additive label (where required)	15			1 (0.8)			2 (0.7)		2 (1.4)	2 (1.4)	4 (5.5)	1 (1.0)		1 (0.7)	2 (1.6)		
Label obscured (not visible in pump)	42	1 (1.1)		2 (1.5)		3 (4.1)	3 (1.1)			7 (5.0)		15 (14.6)	1 (0.9)	2 (1.3)	7 (5.6)		1 (0.6)
Incomplete or incorrect additive label	162	17 (18.9)	24 (18.5)	19 (14.5)	9 (9.3)	3 (4.1)	7 (2.5)	1 (1.2)	6 (4.2)	14 (10.1)		11 (10.7)	2 (1.8)	5 (3.3)	28 (22.6)	2 (1.8)	14 (8.7)
Patient identity (name, hospital number or date of birth)	45	11	3	10						1		8			10		2
Time	57	3	2	6	2		4			3		20		2	9		6
Expiry date	50				4	1				12		25			5		3
Hung by	39		2	2						1		15		3	12		4
Volume	29	1	1		4	1			3	1		16	1			1	
Date	33				1					3		20			7		2
Dose	23			1	1	2	2		1			11			4	1	
Drug name	12	1								1		5	1		4		
Patient’s location (ward)	13	3										9					1
Other	68		24	5			2	1	3			2		2	24		5
Patient identification^a^	116 (5.8%)	9 (10.0)	18 (13.8)	2 (1.5)	9 (9.3)	5 (6.8)		3 (3.6)	11 (7.7)	14 (10.1)	1 (1.4)	2 (1.9)	8 (7.0)	2 (1.3)	21 (16.9)	3 (2.7)	8 (5.0)

Numbers in brackets refer to percentage of infusions with at least one deviation

^a^Deviations are counted per infusion; this figure includes patient identification deviations (i.e. no name band) applied to all infusions for those patients

## Results

Data were collected on 2008 infusions administered or prescribed to 1326 patients between April 2015 and December 2016. Overall, 961 infusions (47.9%) had at least one procedural or documentation deviation. The prevalence of deviations varied considerably among trusts, affecting between 9.9 and 100% of infusions (Table 1). Trusts’ deviation profiles also varied, with some having greater numbers of certain types (Tables 2 and 3).

### Giving set labelling

Deviations in giving set labelling affected 26.8% of infusions. Deviations affected 16.7 to 100% of infusions that required a giving set label across trusts (Fig. 1). Deviations affecting giving set labelling were common at Trusts D, G, H, K and P. Rates of deviation were affected by both the level of detail required by local policy and clinicians’ policy awareness. For example, at Trust D, infusions in all areas except critical care were generally non-compliant; observers only learned that their hospital policy required all IV giving sets to be labelled in the closing stages of data collection, despite being asked to familiarise themselves with relevant policy prior to data collection. In their debrief meeting, observers reported that this requirement was within their peripheral cannula policy and that they had not been able to find it initially. At Trust P, giving sets were only labelled in critical care when this was needed to differentiate between drugs, but their policy explicitly stated that all IV lines must be labelled with the time and date they were connected to the patient; none were labelled with this information. Trust K, which had the most comprehensive giving set labelling requirements, also had a high rate of non-compliance (83.5% of infusions); their policy required all IV lines to be labelled with the name and strength of the medicine, route of administration, diluent and final volume, patient’s name, expiry date and time, and name of practitioner preparing the medicine. Patient Safety Alert 20 [5] does not specify whether or not giving sets need to be labelled.

Eight of the 16 trusts (Trusts B, C, E, I, J, L, M and N) had no trust-wide requirements for labelling giving sets and so had no or low rates of labelling deviations, e.g. Trust B only had policy requirements that applied to critical care. Some trusts required all IV giving sets to be labelled whereas others were more selective. Focus group participants agreed that there were two main reasons for labelling giving sets: 1) to distinguish between multiple giving sets, and 2) to indicate when giving sets need to be changed. Trusts A and O had designed their policy to directly address these points, i.e. “staff had to label giving sets only where more than one was in use and to date them for continuous infusions that would need to be changed.”

### Documentation deviations

**Fig. 2:**
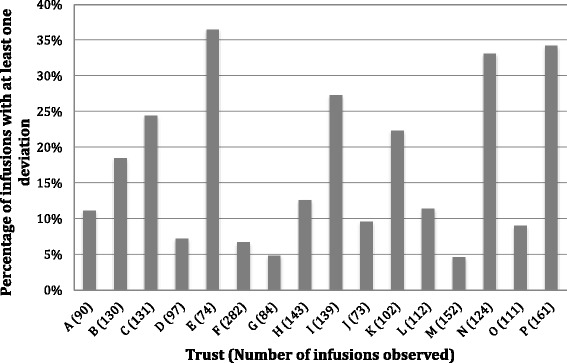
Policy deviations relating to documentation of medication administration

Deviations in documenting IV administration affected 16.7% of infusions, ranging from 4.6 to 36.5% across individual trusts (Fig. 2). Failure to document the start time was the most common problem. Other less frequent but potentially more troublesome issues were discovered during the observations. In some cases, administration was not documented at all. In one case, 20 mmol potassium chloride in a litre of 0.9% sodium chloride was prescribed, but the trust did not stock this formulation. Instead, staff administered two infusion bags of 500 ml 0.9% sodium chloride, with 20 mmol potassium chloride in one of them. However, poor documentation meant it was not clear that this prescription was split across two bags and which was being given first. Patient Safety Alert 20 [5] recommends making a detailed record of the administration as soon as possible after administration but does not give more detailed directions.

### Additive label deviations

**Fig. 3:**
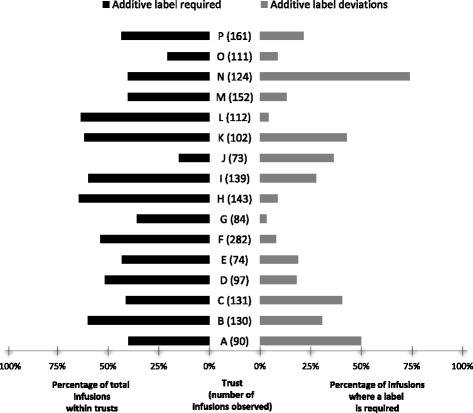
Variation in additive label policy and deviations where a label is required among trusts

Deviations in recording required details on the additive label affected 10.9% of all infusions. Deviations affected 3.3 to 74.0% of infusions that required an additive label across trusts (Fig. 3). Policy requirements affected different proportions of infusions at trusts (Fig. 3). For example, at Trust J, 78% of infusions were standard fluids with no additives, and these did not require a label. Furthermore, not all trusts specified the information required on additive labels in the relevant policy but there seemed to be an implicit expectation in all trusts that nurses should complete parts of the additive labels. Most additive label deviations were considered low risk by observers and focus group participants, such as missing batch numbers for licensed non-biological medications. Trust K had a high deviation rate; their written policy required the most information to be documented on their additive labels: patient’s name, ward/clinical area, drug, final concentration and volume, administration rate, total amount of drug added to the syringe or bag, batch number and details of the medication added [diluent, date prepared, time prepared, expiry date, expiry time, route of administration]. This is more detailed than the Patient Safety Alert 20 [5] recommendations of name of medicine, strength, route of administration, diluent and final volume, patient’s name, expiry date and time, and the name of the practitioner preparing the medicine.

Trust B’s policy required nurses to record batch number on additive labels. However, nurses at the focus group raised objections about the utility of doing this: e.g., for short infusions that would be thrown away after 20 min. They suggested that a better place to record batch numbers (if necessary) would be in the patient’s medical records where this information would be more permanent. One nurse suggested that some medications come with a removable batch number sticker that could be stuck in the patient’s notes. Trust D focus group participants said they had had no detailed additive label requirements written into policy, did not expect the batch number to be commonly completed on the label, and wondered if the labels should be redesigned without the section for batch numbers.

Potentially significant deviations included an additive label only marked ‘DEX’, which referred to dexamethasone but could be confused with dextrose or other drugs; and a completely unlabelled syringe of fentanyl that was in a syringe driver. Other additive label deviations were initially suspected to be medication errors but on further investigation they were solely documentation issues. For example, observers found a 1000 mg bottle of paracetamol infusing into a patient prescribed 675 mg. However, the nurse reported they had removed 325 mg before setting up the infusion, so the patient would receive the correct amount. The observers pointed out that the bottle was not labelled to indicate that this had been done, but the nurses said it was usual practice to remove the excess dose and not label these changes on that ward. Patient Safety Alert 20 [5] makes no recommendations for the process of removing excess dose but does recommend labels are used for medicines prepared in clinical areas and that detailed records of administration are made.

### Patient identification deviations

The percentage of infusions with a patient ID deviation varied between clinical areas: general surgery (2.5%); critical care (2.5%); general medicine (5.1%); paediatrics (9.9%), and oncology day care (10.3%). Patient Safety Alert 20 [5] recommends patient ID and details are checked in accordance with local policy. Safer Practice Notice 11 [10] recommends all hospital inpatients in acute settings should wear ID wristbands.

**Fig. 4:**
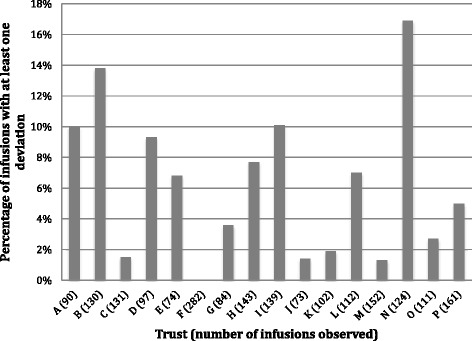
Policy deviations relating to patient identification

The deviation rate relating to ID wristbands was 5.8% overall, ranging from 0.0 to 16.9% across trusts (Fig. 4). Trust F was fully compliant, which may be because this trust had prioritised this area and had been auditing this practice prior to our study. Trust C was the only trust where the policy stated that patients receiving IV infusions in oncology day care were not required to wear ID wristbands. The oncology day care manager at Trust B reported ongoing problems with wristband compliance in oncology day care, although local policy required ID wristbands. She perceived that it was difficult to change staff behaviour and reported technical problems with the printer required for patient ID wristbands.

### Variability in IV flush policies

Most trusts had a patient group direction (PGD) to allow nurses to administer small volume flushes (e.g. 1-20 ml of sodium chloride 0.9%) without a patient-specific medication order. Oncology day care units sometimes used larger flushes that would need a separate prescription if it fell outside the limits of the PGD (e.g. up to 250 ml or 500 ml across a series of infusions). However, at Trust K’s oncology day care unit, such larger volumes were administered without a prescription or PGD; this practice was deemed acceptable by their haematology and oncology care oversight groups. Trust D had an electronic prescribing system that automatically included larger flushes in its chemotherapy regimens, although one flush was observed running but missing from the medication order.

The issue of whether to flush the whole giving set or just the IV access device arose at a number of trusts. For example, at Trust P, a nurse had prepared a 100 ml bag of 0.9% sodium chloride which was not prescribed and was beyond the 20 ml PGD limit, to flush between giving omeprazole and furosemide infusions. The nurse intended to flush the whole giving set to ensure the whole dose was administered and to avoid manipulating the connection with the access device. This was noted as unusual practice by Trust P observers, who reported that the first giving set would usually be detached, the access device flushed and then a new giving set connected for the second drug. However, some focus group participants recognised that this may lead to partial infusions as some of the dose will remain in the giving set. Patient Safety Alert 20 [5] recommends flushing the access device before and after administration.

### Variability in double checking policies

Patient Safety Alert 20 [5] recommends double checking systems, e.g. an independent check or smart pump technology, but it does not go into detail about how these should be done. Different single, double, independent and second checking procedures were required for IV infusions at different trusts. Trusts A and O explicitly permitted single checking for IVs except for specified high risk drugs, specific situations and controlled drugs. For example, Trust O’s policy required staff to double check prior to administration of chemotherapy. In contrast, Trust G’s policy required staff to double check all stages of preparation and administration from cupboard to bedside, although there was acknowledgment in the focus group that this was not always practical. The wording of double checking policies at some trusts implied that this was ‘required’ whereas others seemed more flexible with wording such as ‘where possible’. During the focus group, nurses and pharmacists at Trust P recognised that the wording of their policy was ambiguous, in that the policy was intended to mean that a second clinician signs to confirm that the right patient was receiving the right drug with the correct pump settings, whereas the nursing staff who attended the focus group thought the second signatory was only confirming the contents of the bag or syringe. Trust P pharmacy staff also wanted to move away from the concept of a ‘second checker,’ as this terminology suggested it could be less important and only a confirmatory role, and move towards a ‘second administrator’ who was equally accountable and would be expected to do a thorough independent check. Trust I was the only trust to have a separate detailed appendix to their main policy to specify what an independent double check involved.

## Discussion

Almost one in two infusions had at least one deviation from local policy. Adherence to procedural requirements varied markedly, and it was difficult to make comparisons across sites due to wide variation in local policies, as recently reported in the USA [7]. Most participating trusts had a lower prevalence of deviations relating to additive labels and patient ID than the hospital studied by Husch et al. [6] and fewer additive label and giving set labelling deviations compared to Schnock et al. [7]. We found a higher prevalence of deviations involving patient ID wristbands, compared to 0.2% of infusions recorded by Schnock et al. [7]. The inclusion of oncology day care units and paediatrics in our study exacerbated this difference because of their higher deviation rates compared to other clinical areas.

Patient Safety Alert 20 [5] highlighted the importance of designing adequate and pragmatic procedures for IV safety. Ten years on, this is the first study to investigate the state of IV infusion administration policy and practice in England. High deviation rates at some trusts suggest that policies may have become decoupled from practice. This can be considered as a widening gap between work-as-imagined (policies) and work-as-done (practices) [11]. Observers and staff at some trusts were unaware of the details of their policies prior to taking part in the study. At worst, written procedures can become decontextualised ‘fantasy documents’ [12] that protect the organisation; at best, they can be important resources for action if designed, managed and used appropriately [13].

Overall, variability in policy among trusts suggests that more national guidance and standardisation is needed to help trusts devise appropriate IV infusion policies. We identified clear lack of consensus about whether and how giving sets should be labelled, requirements for additive labels, and procedures for flushing. There was also a wide variation in double checking, with little evidence to guide the development of policy [14–17].

Related to the issue of devising adequate and pragmatic policy is compliance with such policy. Staff sometimes object to requirements that they perceive to be too onerous, or that they find impractical, as illustrated by Trust K having the most prescriptive policy and one of the highest rates of deviations. Staff can face conflict when they are expected to be both efficient and thorough, while policy writers may fail to appreciate the demands of clinical practice [13, 18]. It can also be challenging for practitioners and researchers to determine what is a deviation and what is recommended practice due to the variability in policy between clinical areas and sites. For example, the nurse who planned to flush the whole giving set rather than just the access device was following good practice in one clinical area, but this would have been deemed a deviation in another.

Patient Safety Alert 20 [5] is clear that practical procedures play an important role in safety, as we highlight in the introduction. However, it is difficult to comment on which deviations are safe and which are unsafe, especially when local policy is variable and some requirements are not deemed practical. If a deviation actively contributed to harm it is obviously unsafe, but not contributing to harm in a given patient does not mean that it is ‘safe’. For example, some seemingly safe deviations could have contributed to harm in combination with other circumstances, e.g. a missing ID band could contribute to a patient receiving the wrong drug or a nurse could put up 1000 mg paracetamol intending to stop it early to give a smaller dose, only for an agency nurse to finish the whole bottle in line with standard practice elsewhere.

Annual audits of injectable medicine practice were recommended by Patient Safety Alert 20 [5], but auditing IV infusion administration practice was not common across our participating trusts. Many trusts reported auditing infection control requirements and the prescribing of IV infusions, but not their administration. This may account for staff at some trusts being surprised by the requirements of their own policies and the extent of local deviations. More widespread local audits would reveal issues specific to those trusts, and encourage staff to review issues where policy and practice do not align. These misalignments should be seen as opportunities for organisational learning to reduce the gap between work-as-imagined (policies) and work-as-done (practices) [11]. We would encourage a stance of ‘understanding’ rather than ‘enforcement’ so that practical policies can be devised [13].

A strength of this study is the mixed method approach used to find out both what deviations occurred, and why. However, we did not attempt to analyse whether deviations were intentional or unintentional; this would require interviews with the staff concerned and is an area for further research. A limitation of our study is that there could be differences among pairs of observers, either in their data collection or in their interpretation and knowledge of local policy. We trained all observers to minimise such effects. Some trusts suggested that staffing levels and workload could affect the prevalence of deviations, but we did not collect these data; this could be explored in future studies. Given the variability of local policy discovered in this study, a systematic analysis of hospital policy documents would be fruitful, exploring their variation as well as their adherence to the recommendations in Patient Safety Alert 20. Overall, national efforts are required to identify common standards that balance practicality and patient safety concerns.

## Conclusion

We identified considerable variability in local policies and in procedural and documentation deviation rates. This is particularly concerning given that these risks were highlighted 10 years ago. Some trusts have policies that seem very onerous while others lack policies in certain areas, and are operating outside formal guidance and legislation. Some have policies that are not widely known among staff, or policies that are known but not followed. Standardisation of evidence-based policy is needed as well as better alignment of policies with what is possible in routine clinical practice. Furthermore, active systematic implementation of policy is needed with regular auditing to monitor the alignment between policy and practice.

## Additional file

Additional file 1: ECLIPSE (Exploring the Current Landscape of Intravenous Infusion Practices & Errors): Debrief and focus group guides. This document provides the debrief and focus group guides that were developed as part of phase 1 of the ECLIPSE project. (DOC 46 kb)

## Abbreviations

ID: Identification
IV: Intravenous
NHS: National Health Service (UK)
PGD: Patient Group Directive
TOE: Total opportunities for error

## References

[1] Taxis K, Barber N (2003). Causes of intravenous medication errors: an ethnographic study. Qual Saf Health Care.

[2] Cousins DH, Sabatier B, Begue D, Schmitt C, Hoppe-Tichy T (2005). Medication errors in intravenous drug preparation and administration: a multicentre audit in the UK, Germany and France. Qual Saf Health Care.

[3] McLeod MC, Barber N, Franklin BD (2013). Methodological variations and their effects on reported medication administration error rates. BMJ Qual Saf.

[4] Blandford A, Furniss D, Lyons I, Chumbley G, Iacovides I, Wei L, Cox A, Mayer A, Schnock K, Bates DW, Dykes PC (2016). Exploring the Current Landscape of Intravenous Infusion Practices and Errors (ECLIPSE): protocol for a mixed-methods observational study. BMJ Open.

[5] NPSA. Patient Safety Alert 20: Promoting safer use of injectable medicines. 2007. http://www.nrls.npsa.nhs.uk/EasySiteWeb/getresource.axd?AssetID=60098&type=full&servicetype=Attachment Accessed 25 Apr 2017.

[6] Husch M, Sullivan C, Rooney D, Barnard C, Fotis M, Clarke J, Noskin G (2005). Insights from the sharp end of intravenous medication errors: implications for infusion pump technology. Qual Saf Health Care.

[7] Schnock KO, Dykes PC, Albert J, Ariosto D, Call R, Cameron C, Carroll DL, Drucker AG, Fang L, Garcia-Palm CA, Husch MM. The frequency of intravenous medication administration errors related to smart infusion pumps: a multihospital observational study. BMJ Qual Saf. 2017;26:131–140 10.1136/bmjqs-2015-004465 26908900

[8] Hadi MA, Alldred DP, Closs SJ, Briggs M (2013). Mixed-methods research in pharmacy practice: basics and beyond (part 1). Int J Pharm Pract.

[9] Harris PA, Taylor R, Thielke R, Payne J, Gonzalez N, Conde JG (2009). Research electronic data capture (REDCap)—a metadata-driven methodology and workflow process for providing translational research informatics support. J Biomed Inform.

[10] NPSA. Safer Practice Notice 11: Safer patient identification. 2005. http://www.nrls.npsa.nhs.uk/EasySiteWeb/getresource.axd?AssetID=60032&type=full&servicetype=Attachment. Accessed 9 Feb 2018.

[11] Hollnagel E. FRAM: the functional resonance analysis method: modelling complex socio-technical systems. Ashgate Publishing, Ltd; 2012. ISBN: 978-1-4094-4551-7

[12] Clarke L, Braithwaite J, Wears RL, Hollnagel E (1999). Mission Improbable: Using Fantasy Documents to Tame Disaster. Chicago, IL: University of Chicago Press. Cited in Wears RL, Hunte G. Resilient procedures: Oxymoron or innovation. Resilient Health Care, Volume 3: Reconciling Work-as-Imagined and Work-as-Done. 2016.

[13] Wears RL, Hunte G, Braithwaite J, Wears RL, Hollnagel E (2016). Resilient procedures: Oxymoron or innovation. Resilient health care, volume 3: reconciling work-as-imagined and work-as-done.

[14] Ramasamy S, Baysari MT, Lehnbom EC, Westbrook JI. Evidence briefings on interventions to improve medication safety double-checking medication administration. 2013. https://www.safetyandquality.gov.au/wp-content/uploads/2013/12/Evidence-briefings-on-interventions-to-improve-medication-safety-Double-checking-medication-administration-PDF-888KB.pdf Accessed 4 Aug 2017.

[15] Kellett P, Gottwald M (2015). Double-checking high-risk medications in acute settings: a safer process. Nurs Manag.

[16] Hewitt T, Chreim S, Forster A (2016). Double checking: a second look. J Eval Clin Pract.

[17] Schwappach DL, Pfeiffer Y, Taxis K (2016). Medication double-checking procedures in clinical practice: a cross-sectional survey of oncology nurses’ experiences. BMJ Open.

[18] Hollnagel E. The ETTO principle: efficiency-thoroughness trade-off: why things that go right sometimes go wrong: Ashgate Publishing, Ltd; 2009.

