# Supplementary material for: Procedural and documentation variations in intravenous infusion administration: a mixed methods study of policy and practice across 16 hospital trusts in England
Source: BMC Health Serv Res. 2018 Apr 10;18:270. doi: 10.1186/s12913-018-3025-x (PMC5894189; doi:10.1186/s12913-018-3025-x)
Supplement: Supplementary file 1 — ECLIPSE (Exploring the Current Landscape of Intravenous Infusion Practices & Errors): Debrief and focus group guides. This document provides the debrief and focus group guides that were developed as part of phase 1 of the ECLIPSE project. (DOC 46 kb) [file 12913_2018_3025_MOESM1_ESM.doc]

# ECLIPSE (Exploring the Current Landscape of Intravenous Infusion Practices & Errors):

# Debrief and focus group guides

This document provides the debrief and focus group guides that were developed in November 2015 as part of phase 1 of the ECLIPSE project.

After data gathering was completed at each site it was analysed by the research team and an individual report for that site was developed. This was presented to the site during the debrief and focus group sessions, along with a presentation, which acted as the main driver for what was discussed. We focused on what the individual sites found interesting, relevant and surprising about their own data.

# 1) Debrief guide

## Introduction to purpose/scope

Consent

## General reflections

- What were you expecting to find before starting the data collection? (e.g. were you expecting to find few/lots of errors, any particular kinds of errors, etc.)
- Did anything surprise you?
- What did you learn?
- What important errors did you observe?
  - Why do you consider those ones to be important
- Did you report any incidents as a result of the study?
- Have you identified any changes you would like to make or recommend to be made as a result of the data collection?
- Do you think how we have presented the findings is reflective of what you observed?
- What are the important findings from your perspective?
- Were there any insights that you had that were not captured in our data collection forms and/or report?

## Specific queries about data

Examples:

- Can you explain in more detail how infusions are set up involving both a syringe and volumetric infusion from the same bag?
- In the errors classified as ‘other’ there are quite a few in which the infusion was stopped without a documented reason, what did you think was going on, do you have any theories/views about this, etc?

## Habits

- Did you expect/observe any poor habits?
- Did you expect/observe any good habits?
- What practices are accepted as normal or ok when perhaps they shouldn’t be?

# 2) Focus group guide

## Introduction and overview

Consent

Introductions

Aims/scope/plan of focus group - how the focus group will work

Overview of project

What the data collectors did (data collectors could present this?)/Methodology

Limitations

## Site and summary of point prevalence findings (PPT presentation)

1. Overview of number/proportion of errors and discrepancies overall
2. Medication administration
3. Procedural and documentation
4. Miscellaneous
5. Clinical areas

### General reflection on site findings overall

- Were you surprised by any of the findings?
- What are the important findings from your perspective?

## General themes

- What do you think are the most important safety issues with IV infusions/infusion devices at your hospital/trust?
- Are IV practices a priority/on the patient safety agenda?
- What on-going patient safety initiatives, if any, are there related to IV infusions/infusion devices?
- Do you collect / review any IV-related data for quality improvement purposes?
- What changes would you like to see in either the design of devices or IV practices/policies?

## Site specific questions driven by their results

- For example… patient ID bands in oncology day care // IV boluses given as infusions // what possible reasons are there for such low error/discrepancy rates in X.

## Policies

- How do you feel about local policies and guidelines related to IV infusions?
  - who owns them, are they written well, easily accessible – where are they, well established in practice?
- Are policies enforced? How?
- What policies and or guidelines do you have for double-checking of IV medication? When should the double check take place? When does the double check take place?
- How do policies and/or guidelines (e.g. for labelling tubing or medication) impact on patient safety? How does the absence of policies impact on safety/errors?
- How do you feel policies/guidelines related to IV infusions impact on patient safety?

## Infusions devices

- How is the type of pump/delivery method selected? Are there policies and/or guidelines around this?
  - Are gravity feeds used/allowed? In what circumstances?
  - When are IV boluses given as infusions, and vice versa?

### Smart pumps/DERS sites

- Procurement/implementation
  - Was there a particular rationale for purchasing smart pumps?
  - Was there a rationale for implementing them in some areas and not others?
  - Would you anticipate extending the use of smart pumps into other areas in the future? If not, why not? Etc.
- How were they implemented? Who was involved?
  - What were the challenges and barriers for implementation, e.g. organisational barriers? What impact did these have? How were these overcome, if they were?
- Impact
  - How has the introduction of smart pumps impacted on practice?
  - Have you identified any benefits (i.e. for patient safety)?
  - Have you experienced any negative impacts or unintended consequences?
- Drug libraries
  - How were drug libraries developed? Who was responsible?
  - What is their scope? How comprehensive are they?
  - How are they maintained/updated?
  - What are the policies/expectations around the use of drug libraries? Are people expected to use it? How do you measure/monitor use?
- DERS
  - How were decisions about hard/soft limits made?
  - How do you monitor/respond to overrides, etc.
- Organisationally (if not covered above)
  - Are there broader benefits to the organisation, e.g. in having IV data which is easier to access, in reviewing IV practices prior to implementation
  - What are the organisational costs, e.g. in maintaining drug libraries, reviewing data, etc.
  - What organisational barriers were experienced, e.g. we’ve heard anecdotes from other sites about groups disagreeing about the smart pump implementation and refusing to change their practices?

### Non smart pump sites

- Have you considered, or are you considering introducing, smart pump technology?
  - What do you think about smart pumps technology?
  - What do you think the value of introducing smart pumps would be?
  - What do you think are the key challenges and costs of implementing smart pumps?

## Training

- What training do nurses receive to support their use of pumps and IV practices?
- How do you think training might impact on the types of errors and discrepancies identified in this study?
- Is training standardised across all clinical areas or tailored depending on use?
- Who is involved in developing training? Who runs it? How is it organised?
- Any changes in training as a result of the data?

## Summing up

- Is there anything else that we haven’t discussed that you think is important for us to know? E.g. around training, technology/devices, etc…
- What we’re doing now/next
  - How far we are along with sites in Phase 1
  - The selection of sites and what we will do in Phase 2
  - Workshops with clinicians and other stakeholders in Phase 3
